# Universal Free School Breakfast: A Qualitative Model for Breakfast Behaviors

**DOI:** 10.3389/fpubh.2015.00154

**Published:** 2015-06-11

**Authors:** Louise Harvey-Golding, Lynn Margaret Donkin, John Blackledge, Margaret Anne Defeyter

**Affiliations:** ^1^Department of Psychology, Faculty of Health and Life Sciences, Northumbria University, Newcastle Upon Tyne, UK; ^2^Directorate of Public Health, Blackpool Council, Blackpool, UK; ^3^Directorate of Community and Environmental Services, Blackpool Council, Blackpool, UK

**Keywords:** school breakfast, breakfast behaviors, food insecurity, food poverty, families

## Abstract

In recent years, the provision of school breakfast has increased significantly in the UK. However, research examining the effectiveness of school breakfast is still within relative stages of infancy, and findings to date have been rather mixed. Moreover, previous evaluations of school breakfast schemes have been predominantly quantitative in their methodologies. Currently, there are few qualitative studies examining the subjective perceptions and experiences of stakeholders, and thereby an absence of knowledge regarding the sociocultural impacts of school breakfast. The purpose of this study was to investigate the beliefs, views and attitudes, and breakfast consumption behaviors, among key stakeholders, served by a council-wide universal free school breakfast initiative, within the North West of England, UK. A sample of children, parents, and school staff were recruited from three primary schools, participating in the universal free school breakfast scheme, to partake in semi-structured interviews and small focus groups. A Grounded Theory analysis of the data collected identified a theoretical model of breakfast behaviors, underpinned by the subjective perceptions and experiences of these key stakeholders. The model comprises of three domains relating to breakfast behaviors, and the internal and external factors that are perceived to influence breakfast behaviors, among children, parents, and school staff. Findings were validated using triangulation methods, member checks, and inter-rater reliability measures. In presenting this theoretically grounded model for breakfast behaviors, this paper provides a unique qualitative insight into the breakfast consumption behaviors and barriers to breakfast consumption, within a socioeconomically deprived community, participating in a universal free school breakfast intervention program.

## Introduction

The prevalence of school breakfast has increased considerably in the UK, with a 45% rise in provision since 2008, and current estimates that 85% of schools offer breakfast schemes (1). The UK government recently announced an investment of £3.15 million over 2 years to establish sustainable breakfast clubs in primary and secondary schools (2). From a policy perspective, a fundamental factor is the prevention of hunger in children at the start of the school day (2). With recent reports that almost a third of the UK population experience significant poverty-related difficulties and a quarter of the population live below the accepted standards of living, food insecurity is seemingly an issue faced by a rising number of UK families (3). It has also been reported that approximately 4 million children and adults in the UK consume inadequate diets and one in four adults purposely forego meals, so other family members can eat (3). In food insecure and impoverished circumstances, it has been suggested that the most commonly omitted meal is breakfast (4, 5). It is therefore a reasonable assumption to consider that breakfast behaviors may be detrimentally impacted by high levels poverty and food insecurity.

Furthermore, research has highlighted a greater prevalence of breakfast skipping behaviors among Western populations, with an increased pervasiveness among adolescent females, children from single parent and deprived families, and young adults (6–8). Breakfast skipping has been associated with overweight and obesity (6, 9, 10), in addition to a myriad of deleterious health behaviors including snack food consumption, smoking, alcohol consumption, low physical activity, sedentary behaviors, and weight control behaviors (6, 11, 12). Moreover, research has shown that breakfast omission may lead to more rapid declines in cognitive functions such as attention (13) as opposed to the reported acute effects of breakfast consumption, such as increased alertness, satiety, short-term memory, and mood (14–16).

Research evaluating the effectiveness of school breakfast is still within relative stages of infancy, particularly in the UK, and current findings are inconsistent. To date, positive associations have been reported between school breakfast and academic performance (17), and further research has reported increases in attendance rates in accordance with school breakfast provision (17, 18). Moreover, positive effects of school breakfast consumption on classroom behavior have been reported with observed increases in on-task behavior (19, 20), and teacher reports of reduced hyperactivity and increased alertness (21). Conversely, no effect and negative effects have also been reported in research examining the educational impacts of school breakfast. Results from a recent randomized control trial showed no significant effect of school breakfast on attendance and punctuality in a sample of children from relatively affluent backgrounds (22). Respectively, it has been suggested that positive impacts of school breakfast may be more pronounced among children who are undernourished and/or from lower socioeconomic backgrounds (23). A further randomized control trial found a higher proportion of children participating in school breakfast displayed borderline and abnormal conduct (24). Additional studies have also reported an increase in the prevalence of adverse behaviors such as frustration, rule breaking, and negative interactions with staff among school breakfast attendees (25, 26). Though, it has been suggested that inadequate supervision, and poor organization and infrastructure, within schools and the school breakfast environment, may cumulate in boisterous and disruptive behaviors, thereby confounding research findings (24, 26).

Thus far, the research into school breakfast and breakfast consumption has been predominantly quantitative, with few qualitative studies, particularly in the field of school breakfast. Consequently, there is a dearth of stakeholder voice and experience in the research literature. It is posited that examining the subjective perceptions and experiences of stakeholders may provide important knowledge about the sociocultural impacts of school breakfast. The purpose of this study was to investigate the views, beliefs and attitudes, and breakfast consumption behaviors, among key stakeholders, served by a universal free school breakfast scheme, within a socioeconomically deprived area in the UK. Additionally, the study also aimed to understand the sociocultural impacts of school breakfast from the perspectives of children, parents, and school staff, and to gain an understanding of these impacts at individual, school, family, and community levels. The following research questions provided a framework for the exploration of the phenomenon at the center of this study:

1. What are the views, beliefs, and attitudes toward breakfast among children, parents/carers, and school staff?
2. What are the self-reported breakfast behaviors among children, parents, and school staff?
3. What are the internal and external barriers to breakfast consumption among children, parents, and school staff?
4. What are the views toward the universal free school breakfast scheme among children, parents, and school staff?

This paper addresses an imbalance in the research literature through the presentation of findings from a qualitative theoretically underpinned study into breakfast behaviors and free school breakfast. The paper presents a qualitative model of breakfast behaviors among children, parents/carers, and school staff, which includes breakfast consumption behaviors, and the internal and external factors which influence of breakfast behaviors. In this study, breakfast behaviors included regular breakfast consumption, later breakfast consumption, breakfast skipping, double breakfasting, and breakfast on the way to school. Perceived barriers and influential factors affecting breakfast behaviors in this study were categorized into internal and external factors. Internal factors influencing breakfast behaviors included beliefs about breakfast at home and at school, beliefs about breakfast with the family, and traditional beliefs about breakfast. External influencers of breakfast behaviors primarily included socioeconomic factors including poverty and food insecurity; work, educational, household, and family commitments and responsibilities; and family structure.

## Materials and Methods

Data were collected from a purposive sample of children, parents/carers, and school staff recruited from three primary schools located in an area of high socioeconomic deprivation within the North West of England, UK. The demographic characteristics of each school are provided in Table 1. All schools had a higher proportion of the populace claiming working age benefits than the proportion of the population across the whole of the North West of England (19%) and England overall (15%). School 1 had a particularly high proportion of children entitled to free school meals (61.8%), three times higher than the proportion eligible in the other two schools. The proportion of those claiming working age benefit within the local ward that School 1 was located was also higher than the subsequent two schools. While all three school wards rank highly as “most deprived” on the Indices of Deprivation (27), School 1 ranked highest on income, employment, health, and housing deprivation indicators.

At the time the data were collected, all three schools were participating in an established universal free school breakfast scheme, funded by the local council, which had been operating for over 1½ years. Information pertaining to the breakfast model adopted by each school is provided in Table 2. Fifteen predominantly white British children (mean = 9:0 years; range 6; 2–10; 8 years; 8 female/7 male) were recruited from the three participating schools. Parents/carers of participating children were also invited, including a sample of 16 predominantly white British parents/carers (mean = 41; 10 years; range 26; 8 to 65; 2 years; 12 female/4 male). Finally, 16 predominantly white British female school staff (mean = 41; 11 years; range 24; 0–60; 2 years), from three participating schools, were recruited, including a sample of teachers, teaching assistants, school breakfast staff, and managers. Full ethical consent was approved by the University Ethics, Northumbria University Newcastle.

**Table 1:** **School characteristics and school area demographics**.

Schools	School demographics^a^					School and local area demographics^b^	
	Pupils on role (*N*)	School type	Pupils with special educational needs (%)	Pupils with English as an additional language (%)	Pupils entitled to free school meals (%)	All people of working age claiming a key working benefit (%)	White British (%)
1	453	Community aided	13.5	8.6	61.8	34	94
2	486	Voluntary aided	5.1	Supp^c^	20.6	22	96.1
3	210	Voluntary aided^d^	4.5	15.7	20.0	24	94.9

^a^Information taken from <http://www.education.gov.uk/>

*^b^Information by ward taken from <http://www.neighbourhood.statistics.gov.uk>; state benefits including jobseekers allowance, incapacity benefit or severe disablement allowance, disability living allowance, income support*.

*^c^Information has been suppressed because underlying figures are too low*.

*^d^Since the study was conducted, the school has become a sponsor led academy*.

Semi-structured interview schedules were developed using Ajzen’s theoretical concepts of the Theory of Planned Behavior (28). Open ended questions examining sociocultural beliefs, views, attitudes, subjective norms, and behavioral intentions toward breakfast and past breakfast behaviors were incorporated into the interview *pro forma*. A total of eight small focus groups were undertaken with children, parents, and school staff, and 29 interviews were undertaken with parents and school staff. Discussions were guided by the interview schedules and concluded when participants felt they had nothing more to contribute. Member checks were utilized throughout to increase validity. The interviews and focus groups were audiotaped using a Dictaphone recorder and transcribed verbatim for subsequent analysis.

Data transcripts were the main unit of analysis and each transcript was subject to an in-depth reading to obtain a holistic sense of the data. Grounded Theory was utilized as a framework for the analysis, in particular Strass and Corbin’s model (29), open coding, axial coding, and selective coding. During the first stage of analysis, all the data were imported into Nvivo 10 for ease of access and organization. This was ensued by an inductive process involving a detailed reading of the transcripts, which were tagged with memos and organized into codes relating to the conceptual ideas that emerged. Subsequently, during a deductive process, the codes were subject to a comparative analysis where relationships were identified and codes were organized into a smaller number of larger themes. The final stage of analysis involved a thematic synthesis between groups, i.e., children, parents/carers, and school staff, resulting in a qualitative model for breakfast behaviors. Approximately 10% of the data transcripts were coded by a second researcher, with good inter-rater reliability (Cohen’s Kappa .7) demonstrating considerable agreement between coders (30).

**Table 2:** **School breakfast models**.

Schools	School breakfast models^a^			
	School breakfast timings^b^	Food served	Food serving model	Milk and fruit served at break-time
1	8:15 a.m.	Toasted bread products, water	Self-serve	Yes
2	8:50 a.m.	Bread products, fruit, yogurt, water	Self-serve	Yes
3	8:30 a.m.	Bread products, fruit, yogurt, water	Individualized servings	Yes

*^a^Information provided by schools at the time of the study*.

*^b^Schools 1 and 3 provided breakfast before the start of the school day. School 2 served breakfast at the start of the formal school day*.

## Results

**Figure 1:**
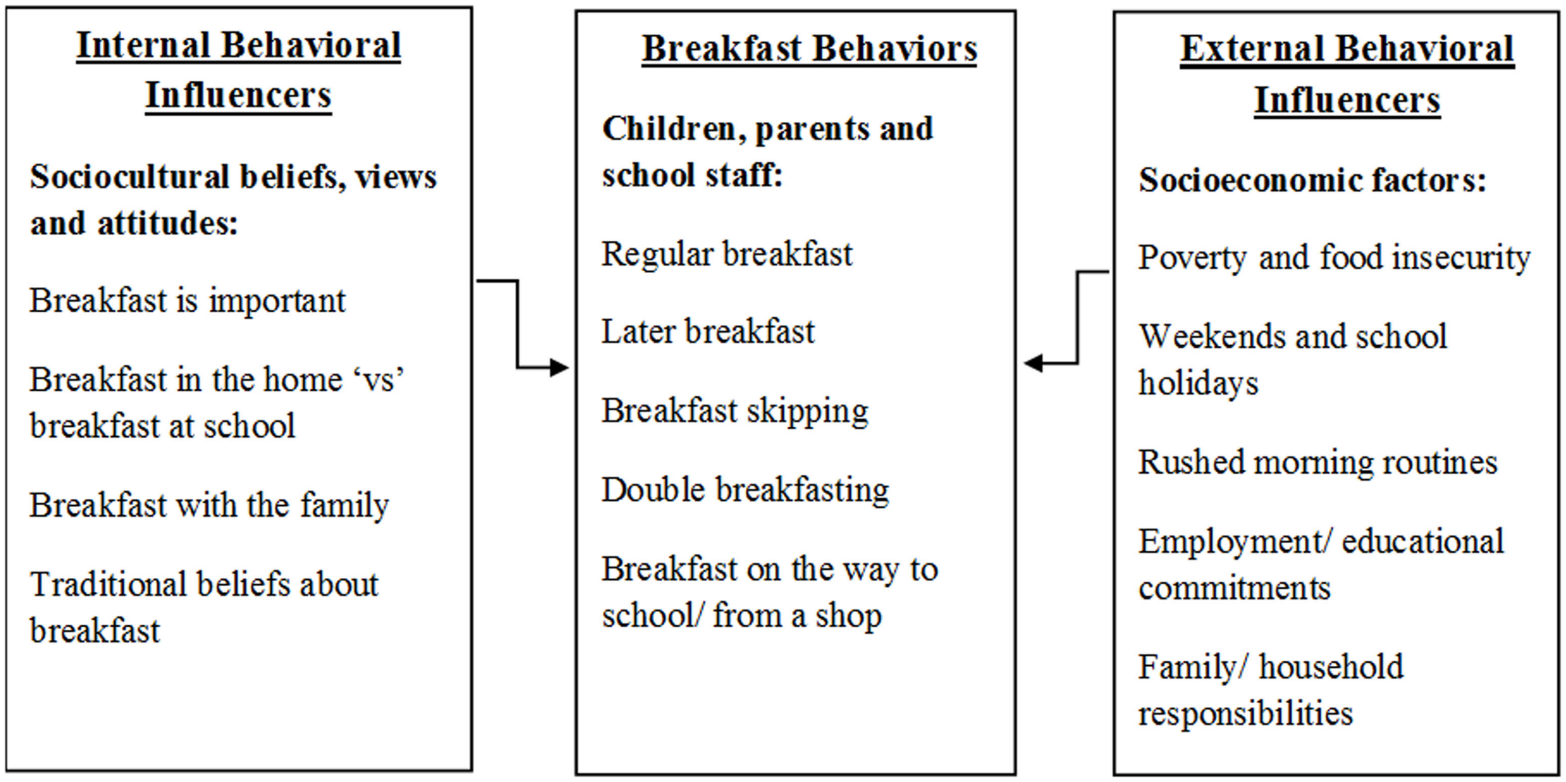
**Qualitative model for breakfast behaviors**.

The qualitative model of breakfast behaviors, which is visually represented in Figure 1, consists of three domains directly relating to breakfast behaviors, and the internal and external factors that are perceived to influence these behaviors. The primary domain entitled, “*Breakfast behaviors*,” includes themes relating to regular breakfast consumption, later breakfast consumption, breakfast skipping, double breakfasting, and breakfast on the way school. The domain entitled, “*Internal factors influencing breakfast consumption*,” encompasses themes relating to sociocultural beliefs, attitudes, and views about breakfast and the breakfast environment. The domain entitled, “*External factors influencing breakfast consumption*,” comprises of themes relating to the socioeconomic factors that are perceived to impact on breakfast consumption behaviors including, poverty and food insecurity, employment and educational commitments, family and household commitments, and family structures. The three main sections below present these key themes under each domain as represented in the visual model (Figure 1). The findings are presented in narrative format with verbatim quotations from participants in order to represent and retain stakeholder voice and experience.

### Primary domain: Breakfast behaviors

This primary domain is comprised of prevalent themes relating to the reported breakfast behaviors among children, parents, and school staff. Themes are presented under key headings relating to regular breakfast consumption, later breakfast consumption, breakfast skipping, double breakfasting, and breakfast on the way to school.

#### Regular Breakfast Consumption and Later Breakfast Consumption

Regular breakfast consumption and later breakfast consumption were key themes under the domain of breakfast behaviors. Children, parents, and school staff reported eating breakfast regularly; however, reports of eating breakfast later in the morning were primarily relating to adults, namely parents and school staff.

Those who reported regular breakfast consumption, i.e., every day, described the breakfast meal being a habitual part of their daily routine, in the same respect as other routine behaviors such as washing, brushing teeth, and getting ready, “*I think it’s just in your routine, it’s just part of what you do, you get up, have a shower, get your breakfast*” (Parent/carer). Moreover, among those who reported eating breakfast every day, breakfast was considered to be part of the family’s routine, suggesting that habitual breakfast consumption may typically be adopted across the whole family unit. Additionally, for those who reported that they ate breakfast every day, illness or sleeping late were perceived to be valid reasons for skipping the breakfast meal, “*Only if we’re ever ill. No other reason that we wouldn’t have it*” (Parent/carer), suggesting strong behavioral intentions toward eating breakfast every day.

Some parents/carers and school staff reported that they consumed breakfast regularly, but later in the morning. This was perceived to be due to a lack of appetite and professed feelings of fullness early in the morning. In some instances, these feelings of fullness were due to late night eating, “*I probably eat a bit late at night time, maybe so I’m still a bit full in the morning*”(Parent/carer), suggesting a potential association between late night eating and breakfast consumption later in the morning. Furthermore, rushed mornings were also cited as reasons for consuming breakfast later in the morning, with work and educational commitments, household and family responsibilities, and families with multiple or young children considered to be key contributors to chaotic mornings, *“I’m up and I don’t want anything to eat at 6’o clock in the morning, so I won’t”* (School Staff). It was perceived that these commitments and responsibilities in the morning contributed to a lack of time resulting in an inability to eat breakfast early in the morning.

Participants who reported eating breakfast regularly described consuming a variety of breakfast foods and beverages including, cereals, bread products, fruits, dairy products, tea, and coffee, “*I’ll go home now, I’ll have a couple of slices of toast with my marmite and off I go to work and I’ll be fine*” (Parent/carer). Consumption of variety of breakfast foods was consistent across those who ate breakfast early and later in the morning. In addition, those who ate breakfast later also reported that they did consume breakfast beverages early in the morning, including probiotic drinks, tea, coffee, and water, “*I tend to drink, the odd time I’ll have a cuppa tea, but I drink a lot of water*” (Parent/carer), and thereby were actually consuming something early in the morning. Furthermore, minority of participants, who reported they consumed breakfast later, described eating foods high in fat for breakfast, “*I have a fry up every day, and then do my work*” (Parent/carer). It could be suggested that this may be associated with increased hunger later in the morning, although this was not reported.

Parents/carers, who reported consuming breakfast later, expressed concerns about the potential detrimental impact of their own breakfast behaviors on their children’s behaviors, “*I suppose it’s not setting a good example cause they don’t see you doing it*” (Parent/carer). This may indicate that these parents/carers perceived eating breakfast later in a negative light, and were concerned about their children being aware of these behaviors, and thus potentially modelling the behaviors. These concerns may have some validity in light of some children’s reports, indicating an awareness that parents/carers were eating breakfast later in the day, *“My mum sometimes doesn’t have any, but [has breakfast] when she gets home”* (Child). While some children were aware that their parents/carers ate breakfast later, there were no indications that these children were modeling these behaviors during the school week reported in this study.

#### Breakfast Skipping

A further theme, under the domain of breakfast behaviors, was breakfast skipping. Breakfast skipping was discussed by parents, children, and school staff, both concerning self-reported behaviors and concerns about children skipping breakfast.

Corresponding with those who ate breakfast later, professed feelings of fullness and lack of appetite, sometimes due to late night eating, were also cited as reasons for skipping breakfast completely, “*I just think oh I’m not hungry at the minute, I don’t fancy it at the minute*” (Parent/carer); “*I have had times where my children’s like, ‘well I’m not hungry, I don’t want anything*”(Parent/carer). Likewise household, work, educational and family commitments, contributing to rushed morning routines, were also reported a key factors in the prevalence of breakfast skipping.

In sharing their experiences of breakfast skipping, some parents/carers and school staff described instances where skipping the breakfast meal had made them feel fatigued, nauseous, and ill: “*I feel a bit yucky and dizzy and I’ve been having a lot of headaches*” (Parent/carer); “*I’ve missed my breakfast when I’m in a hurry and you’ve just got no energy, nothing”* (School Staff). A minority of children, who reported skipping breakfast in the past, described lacking energy, feeling fatigued, and worrying about having to wait until lunch time for some food to alleviate their hunger, “*When I don’t have my breakfast I have to wait all that time until dinner time and it just makes me real tired like I’m gonna fall to sleep*” (Child). There were concerns about the impacts of the negative consequences of skipping breakfast on children’s learning, with school staff reporting instances where children had skipped breakfast and as a result were unwell at school, “*They can be more sluggish, they can lack, I think it’s the headache, it’s everything that comes with it, but grumpiness and they’re lethargic”* (School Staff). School staff reported that children who missed their breakfast meal would typically lack energy and display detrimental behaviors such as negative mood and frustration in the classroom.

In the context of children skipping breakfast, suspected instances of skipping breakfast, as a means of weight control, were reported by school staff at one school, specifically concerning a minority of older primary school female children. Staff from this school reported instances where they had discovered a minority of older female children skipping breakfast and attempting to skip school lunches, “*Skipping breakfast and then cause they’re on school dinners trying to stop eating*.” Some parents/carers and school staff considered that these eating behaviors, among young girls, may be attributed to imagery in the media and changing body shapes due to the onset of puberty.

Girls already year 6 being body conscious. They don’t like that they’re changing cause if you think about cartoons, all cartoons have got little girl figures and by the end of year 6, some of them haven’t got their little girl figure, they’re changing, and they don’t like it cause they’re normal, and normality isn’t often shown in the media is it? (School Staff)

In this school, it was reported that staff and parents/carers worked together to identify children who were skipping breakfast, so support and monitoring could be implemented for these children at home and school.

#### Double Breakfasting and General Overeating

Further themes, under the domain of breakfast behaviors, were reported instances and concerns about double breakfasting and general overeating in the morning among some children, due to the additional free breakfast provided at school.

Disconcertingly, some school staff expressed concerns that certain children were eating breakfast foods across multiple locations and felt that consequently they were overeating, “*Other children who eat at home, go to early birds, have their breakfast there, come in school have their breakfast there and by then break might have a piece of fruit”* (School Staff). While some school staff did confirm that the majority of children would advise them that they had already consumed breakfast at home, they also claimed there were a minority of children who they felt were overeating in the mornings, “*A lot of them do just say I’ve had it already and they’re fine and they know, but some of them do eat loads*,” (School Staff). Furthermore, some school staff conveyed concerns about the potential of double breakfasting contributing to an obesity problem among children, “*In the wider scheme you’ve got obesity issues*.” (School Staff).

These concerns may be valid in light of a minority of children reporting that they frequently ate more than one breakfast, “*Two and one basically, maybe three if I’m lucky*” (Child), and perceptions that two breakfasts may be healthier than one, “*If you have two breakfasts it might make you healthier than just one*” (Child). Moreover, some children reported that they had observed a minority of their peers overeating at school breakfast, “*Some people just get one thing and then another and then another and then another*” (Child). Reassuringly, other children maintained that they moderated their breakfast consumption in school; only consuming small items and declining breakfast foods at school when they had already eaten similar items at home, “*I had toast and today it was toast but I just couldn’t fit any more toast*” (Child). Though, others confirmed that they did not consume less breakfast foods at home to counterbalance for breakfast eaten at school, “*No I have the same about at home*” (Child). Other children reported that they consumed breakfast at home every day, yet only consumed breakfast at school sometimes, “*I sometimes eat it and sometimes don’t*” (Child). On these occasions, children claimed they were more likely to eat additional breakfast foods at school if it was foods they liked, “*I have a lot of stuff for breakfast to get me energy up and I just come into class and I can’t eat anymore but I just can’t resist pancakes*” (Child).

Furthermore, in certain circumstances, school staff provided justification for children eating breakfast foods at home and at school; for example where children walked to school, “*His parents say he has a breakfast, but for him all that energy he’s used walking he’s already worked his breakfast off”* (School Staff). In addition, some school staff also reported that most children were efficiently managing their breakfast food intake at home and school, and would stop eating once they were satiated, “*Mine last year were quite good at saying, no I’ve already had breakfast I’m not hungry, they’d only really eat when they were hungry”* (School Staff). Parents/carers, who provided their child with an early breakfast due to work commitments, also claimed they were provided with assurances that their child would not be hungry through the provision of an additional school breakfast, “*Even though he has had a small breakfast at like 8’o clock it puts him over till dinner time*” (Parent/carer). Encouragingly, some parents/carers did claim to moderate their children’s breakfast consumption at home by providing a reduced amount of breakfast food when their child would be eating breakfast at school, “*Obviously I control the amount he has in the house, normally I would give him a certain amount, I give him slightly less than that*” (Parent/carer). Interestingly, it was also noted that the negative stigma, associated with not providing children with breakfast at home, may be an attributable factor in the prevalence of parents/carers, who have continued to provide breakfast at home, in the knowledge that they will be consuming breakfast at school, “*I think some parents as well they don’t to be seen, do you know like, you can’t afford to give your kids breakfast”* (Parent/carer).

#### Breakfast on the Way to School

The final themes under the domain of breakfast behaviors were relating to children eating breakfast on the way to school, and/or purchasing food, often high in fat and/or sugar, for their breakfast from a shop. Some school staff claimed to have observed certain children arriving at school with snack food items, such as crisps and chocolate for their breakfast, “*Snack foods like crisps and this is what we see children walking to school with chocolate bars”* (School Staff). In addition, some children claimed that their peers received money from parents/carers to purchase food for breakfast from the shop on the way to school, “*A lot of children get money from their parents and they can just go to the shop and buy sweets for their breakfast*” (Child).

Rushed morning routines were considered to be attributable factors, among some school staff, contributing toward the prevalence of children eating snack foods for their breakfast on the way to school, and/or purchasing food from a shop in the mornings, “*It’s the rush, getting everyone ready for school and then maybe just sent out the door and a lot of them do come in eating an apple, drinking a fizzy drink”* (School Staff). It was also supposed that the prevalence of children arriving to school with snack food items may be attributed to the high levels of socioeconomic deprivation in the local area, “*We’re in a deprived area so a lot of children come to school without breakfast or with a chocolate bar in their hand or a bag of crisps in their hand”* (School Staff).

Promisingly, it was considered among school staff that the universal free school breakfast scheme was providing a positive intervention, and reducing instances of children eating snack foods on the way to school, “*A lot of the children that we have in breakfast club were the ones walking to school with a bar of chocolate in their hand or a little fist of biscuits but now they’re not”* (School Staff); and/or purchasing snack foods for breakfast from a shop, “*Children don’t stop off at the shop the same cause they’re coming here”* (School Staff).

### Secondary domain 1: Internal factors influencing breakfast consumption

This secondary domain encompasses themes relating to the internal factors, which were perceived to have an influence on breakfast consumption behaviors among participants. These internal factors are presented under themes relating to the perceived importance of the breakfast meal, beliefs surrounding breakfast at home vs breakfast at school, and traditional beliefs about breakfast.

#### Perceived Importance of the Breakfast Meal

Breakfast was perceived overwhelmingly among the majority of participants as the most important meal of the day, “*I think it’s really important. You need a good breakfast before you go out. It is the most important meal of the day*.” (School Staff). Breakfast was believed to be of significant importance due to its timing; typically after a long period of sleep and fasting, “*That’s the longest spell you’ve been without food so I think yeah breakfast is important”* (School Staff). In this context, eating breakfast was thought to be associated with the stimulus of the body’s metabolism and increasing feelings of energy and alertness, “*It kick starts your day, it starts off your metabolism and it’s, it wakes you up”* (School Staff). Moreover, children also recognized breakfast as being of vital importance for the energy required for school activities, “*It can keep people’s energy up and you can run around more often instead of being slow and not playing a lot*” (Child).

It was considered among participants that breakfast consumption had positive impacts on learning and classroom behaviors for children, via perceived benefits on health, well-being and cognition, “*It keeps them healthy and people get ready to learn*” (Child); “*It is brain food, they’re going to be more content going to the classroom and learning*” (Parent/carer). Comparably, it was also deemed among some participants that skipping or missing the breakfast meal may result in negative impacts on learning, “*It feeds the brain and so you know if they’ve not had anything to eat first thing the morning, there are not motivated, they’re sluggish*” (Parent/carer); and classroom behavior, “*The bad behavior creeps in, because they just don’t know what to do and then they’re not concentrating so they will have annoyed the person next to them”* (School Staff). One child, who reported skipping breakfast in the past, claimed that missing breakfast had resulted in disturbances in attention and short-term memory, “*You just forget everything that you’re doing and the teacher has to stop helping other people and go through it again*” (Child).

#### Sociocultural Beliefs About the Breakfast Environment – Home vs School

This sub theme was relating to perceptions among participants about the advantages and disadvantages of breakfast at home vs breakfast at school. There was a prevalent perception among participants that home was the best environment for children to eat their breakfast meal. A primary factor among parents/carers and school staff, underpinning the belief that the home was the best environment for children to consume their breakfast, was the perception that breakfast provision for children was a parents’ job, “*I just think it’s your job isn’t it?*” (Parent/carer). Irrespective of the universal free school breakfast scheme, some parents/carers regarded the practice of replacing breakfast at home with breakfast at school would lead to complacent parenting, “*Once you stop actually giving them breakfast at home you’re starting on a lazy path there. Oh I won’t bother, let school do it*” (Parent/carer). Moreover, these parents/carers were resistant that school breakfast provision would in no way prevent them from providing their children with breakfast at home, “There’s no way I’d let anyone else feed Katy*” (Parent/carer); irrespective of the universal free school breakfast provided to their children at school, “*When we thought they would have breakfast at school, it didn’t stop us giving em breakfast at home*” (Parent/carer).

Motivations for having a preference toward children eating breakfast in the home included parents/carers ability to exercise more control over what children were eating at home for their breakfast meal, and it provided them with assurances their child would not be hungry while at school, “*Knowing that their tummy’s full and then you’re not gonna worry when they’re at school that they’re hungry. I know that she’s had enough*” (Parent/carer). Additionally, certain parents/carers expressed concerns about their children not eating breakfast at school, “*I can see what they’re eating and make sure that they are actually getting breakfast whereas if I just left it to school they might just come in and think oh I can’t be bothered. I’m not in control of it*” (Parent/carer). In some of these cases, it appeared that there was a perceived lack of trust that children would actually eat breakfast at school, in addition to a reluctance to relinquish control over their child’s breakfast consumption.

However, it was also acknowledged among participants that breakfast at home was not always practical due to lifestyle factors such as work commitments and family structures, “*If your mum and dad’s rushing out to work or they’ve got other siblings to take to school, you can’t always eat in your home*” (Parent/carer). There was an acceptance that in these circumstances, school breakfast was preferable as opposed to a rushed breakfast, breakfast eaten on the way to school, or even no breakfast, “*Ideal world it would be at home, but if that’s not the option or if they struggle then I agree that it should be somewhere else rather than just as they’re walking*.” (School Staff). Moreover, the provision of a “healthy” breakfast meal was also regarded as a significant factor beyond merely the environment where breakfast was served, “*As long as the person’s getting a decent healthy, well fairly healthy anyway meal, it doesn’t really matter*” (Parent/carer).

#### Sociocultural Beliefs about Breakfast with the Family

A further theme under this domain comprised of beliefs about breakfast with the family. Breakfast with the family was perceived to be of significant importance among certain participants and a key reason for preferring breakfast at home, “*Some families want to spend some time with them when they have their breakfast before they go to school*” (Child). Some parents/carers considered breakfast a time for family communications, “*You’re sat bonding with your kids sat having something to eat*” (Parent/carer). Moreover, some children reported that they felt more comfortable eating breakfast at home with their family, “*You’re eating with your mum and dad at home so it makes you feel even more comfortable*” (Child).

Additionally, there was also prevalent perception among adult participants, namely parents/carers and school staff, that breakfast should be served and eaten at a table, preferably with the family, “*In my house all together at a table, nowhere else, TVs not on, phones aren’t allowed at the table*” (Parent/carer). Eating breakfast at the table was associated, among these participants, with “good family values,” and a decline in these traditions was perceived as a general decline in family values, “*This is what goes wrong in our days with people, they don’t sit at a table and eat their meals together*.” (School Staff).

While there was a preference toward eating breakfast with the family among some participants, in some of these instances household commitments meant that parents/carers were not always eating with children, “*My two little boys sit and they have their breakfast and I have mine and we like chat, well I’m actually getting on with jobs*” (Parent/carer). Likewise, some parents/carers claimed that rushed morning routines meant that eating together as a whole family was often unrealistic, particularly for large families and families with young children, “I know it’s hard for some parents, I mean I’m rushing about and it’s only just myself and Suzy*. Especially if they’ve got babies and little ones to look after” (Parent/carer). Moreover, as parents themselves, some school staff also reported that work commitments also prevented them from eating breakfast at home with their children, “*Everybody’s got busy lives haven’t they? I don’t do it at home with my children*.” (School Staff).

There was an acknowledgment, among participants, that breakfast at school was a positive alternative to a rushed breakfast or no breakfast at home, “*Obviously it would be better at home to sit having conversations with maybe your siblings and your mum and dad but if that’s not possible, then to bring them somewhere – I think it’s best for them rather than having nothing*.” (School Staff).

### Secondary domain 2: External factors influencing breakfast consumption

This final secondary domain incorporates themes relating to the external factors, which were perceived to have an influence on breakfast consumption behaviors among participants. These themes are relating to poverty and food insecurity, food insecurity during weekends and school holidays, work and educational commitments, family and household commitments, and family structure.

#### Poverty and Food Insecurity

As evident from the deprivation measures provided in Table 1, the local area, in which the schools participating in this study were located, experiences multiple levels of deprivation, “*This catchment area that’s what it come down to, it’s a deprived area for many reasons*.” (School Staff). It was perceived that rising levels of poverty in the area had resulted in families experiencing increased food insecurity, “*It’s such a poor deprived area, and people just can’t afford [food]. There’s food banks in the area. There were never food banks when I first moved here*” (Parent/carer). It was considered that these rising level of food insecurity were having a detrimental impact on breakfast consumption behaviors among families experiencing poverty, “*In this catchment area, we’re in a deprived area so for whatever reason a lot of children come to school without breakfast*.” (School Staff). It was supposed that the poverty experienced by some families in the local area may be impacting on parents’/carers’ ability to afford the cost of breakfast foods, “*We are quite a poverty stricken area and it perhaps doesn’t seem a great deal of money to somebody that’s got a little bit in their pocket but when you have nothing, cereal, toast, it can mean you know going without*.” (School Staff).

Some school staff claimed to have genuine concerns about families experiencing high levels of food insecurity, and worried about the subsequent impact on children, such as missing meals and consuming low quality foods. This was a particular concern for large families with multiple children to feed, “*There are families of concern that we think we know they’re not going to have that breakfast when they get home and they won’t have the quality. When there’s lots of them, big families, you know it’s the cost, especially during [school] holidays*.” (School Staff). Staff from one school reported providing assistance to families experiencing food insecurity in the past via a local food bank. Moreover, some school staff expressed additional concerns about some families experiencing working poverty, “*It tends to be the working parents that struggle, the working parents and the ones who can’t claim for anything*.” (School Staff). There were specific concerns about those in low paid and multiple employment and those living in private rented and/or multiple occupation accommodation, “*Lower paid jobs and they’re on the breadline, they’re in rented accommodation and multiple occupation. Cause some do two jobs*.” (School Staff). It was perceived that these parents/carers may be doubly disadvantaged by low incomes and a lack of eligibility for benefits such as free school meals.

Reassuringly, it was perceived that the universal free school breakfast scheme was contributing to the mitigation of food insecurity in the community. It was supposed that the provision of a breakfast meal for children provided a small level of financial assistance for families and reassurance for families experiencing food insecurity, “*I think it just takes the pressure off them and it just gives them the knowledge that the child is gonna come to school, have something to eat to set them up for the day till lunch time*” (Parent/carer). The universal element of the free school breakfast program was considered a significant feature for those parents experiencing working poverty, and potentially not eligible for free school meals.

#### Food Insecurity During Weekends and School Holidays

A further prevalent theme under this domain was food insecurity specific to weekends and school holidays. Disconcertingly, a minority of children did confide that they experienced food insecurity during weekends and school holidays, “*Sometimes I don’t have any*…*because sometimes on weekends there isn’t any breakfast things in, because erm the food are only in little boxes and erm there’s isn’t any left*” (Child), implying that they sometimes missed breakfast during these periods because there were no breakfast foods in the home. Moreover, some school staff reported that they were aware of families who were experiencing amplified food insecurity during weekends and school holidays, because of the additional food costs for breakfast and lunch, particularly during the longer summer holidays, “*You’re thinking if this is the beginning of the summer holidays, they’ve got 6 weeks*.” (School Staff). There were concerns among school staff about particular families’ access to adequate food during weekends and school holidays and specific anxieties about the impact on children experiencing food insecurity during these periods. “*You know certain children and you think what’s going to happen to them over the holidays. You’re worried, are they getting fed, and probably the answer is no, they’re not and it must be hard for children as well*.” (School Staff).

School staff from one school reported organizing food provision assistance to families during school holidays via a local food bank. Moreover, some school staff considered there was a definite need for breakfast provision during school holidays to alleviate food insecurity for families during these difficult periods, “*I think for some children, some families there is a definite, definite need, you know we’ve had to give food parcels out and things*.” (School Staff). However, it was also acknowledged that the additional provision would be difficult due to perceived funding issues and sustainability of a holiday feeding program, and the potential negative stigma it may attract, “*How would we cope during the holidays, and if we did there would be a stigma*.” (School Staff). School staff had concerns that the school would find it difficult to offer food provision to children during the school holidays due to these issues.

Further concerns for families, during weekends and school holidays, in addition to the costs of providing food for children that they would normally receive at school, were the extra childcare costs for parents/carers with work or educational commitments, “*Childcare is an issue, they can’t all pay £25 for whatever*”(school staff). Living in a seaside town reliant upon seasonal local tourism, it was considered that the issue of childcare was amplified for some working parents/carers during the school holidays, specifically those in employment where they were unlikely to be authorized leave during busy school holiday periods, “*Holidays are a killer, a lot of them are working in the holidays and they can’t have the time off in the summer, so for us as a seaside town*” (School Staff).

#### Work/Educational Commitments and Family/Household Responsibilities

Work and educational commitments were further themes under the domain of external factors, which were perceived to influence breakfast behaviors. It was reported by some participants that work and educational commitments meant that morning routines were often rushed, and consequently had an impact on the family’s breakfast behaviors, “*There’s a lot of chaos in my house in a morning. You know everyone is busy getting ready for work*” (Parent/carer). Parents/carers and school staff claimed that combinations of work and/or educational commitments, and household and family responsibilities, often had a detrimental impact on their breakfast behaviors, “*Yesterday I didn’t [eat breakfast]. I’ve got to go to work in the afternoon. I need to make sure that I’ve got everything done by lunch time get him ready for school*” (Parent/carer), resulting in them skipping breakfast or eating breakfast later.

Encouragingly, in schools where breakfast was served before the start of the formal school day, it was considered that free childcare in the morning, in addition to a free breakfast alleviated morning routines for parents/carers with educational commitments, “*I’ve got to be at college for 9 in the morning, so that helps me drop them off before*” (Parent/carer), or work commitments, “*If someone has a 9 to 5 job they’re going to struggle to get them up, ready and fed and brought to school*” (Parent/carer). Moreover, it was also perceived that the provision of free school breakfast before the start of the school day alleviated the financial costs associated with fee bearing breakfast clubs, which were previously utilized by working families, “*If they’ve got work and they don’t have to pay for breakfast club. They can drop them off a little bit earlier and then get to work and I know some parents that do that*” (Parent/carer).

#### Large Families and Families with Young Children

Additional themes under this domain were the impacts of having multiple or young children on morning routines and breakfast behaviors, “*It could be a struggle you know three children and a baby*” (School Staff). One parent, with a very young child, claimed they often skipped breakfast, due to a lack of time with the extra caring responsibilities in addition to the usual household and family responsibilities, *“I’ve got my hands full. I’m up and I get everything ready. Usually I try and do it [eat breakfast] before her bottle, but if not then I’d quickly do theirs and I’d just leave mine and that’s when I’d forget to have it* (Parent/carer). It was considered that the additional responsibilities associated with having very young or multiple children may have a detrimental impact on breakfast consumption behaviors, in that parents/carers perceive they do not have time for their own breakfast and consequently skip breakfast or eat breakfast later.

Reassuringly, it was perceived, among participants, that free school breakfast provision alleviated rushed morning routines for larger families, “*I know it can be quite a rush in the morning when you’ve got lots of children, so to bring them in and know that they’re here and they’re ready*” (Parent/carer), in that the provision of breakfast for children meant that parents/carers did not have to provide a breakfast at home, and thereby saved time in the mornings. It was also considered that free school breakfast eased some of the financial pressures associated with the costs of providing breakfast food for larger numbers of children, “*I think it helps them, a million percent, especially if they’ve got a few children*” (Parent/carer). Moreover, it was also apparent that free school breakfast eased morning pressures for parents/carers with young children, “*My mum has a baby now so it and now she needs, she doesn’t have much time to do our breakfast so I think it’s helping her that we go to breakfast club*” (Child).

## Discussion

The current study set out to examine the sociocultural beliefs, views and attitudes toward breakfast, and the self-reported breakfast consumption behaviors, among key stakeholders at the center of a council wide universal free school breakfast initiative, within an area of high socioeconomic deprivation, in the North West of England, UK. A grounded theory analysis of the qualitative data, collected during semi-structured interviews and small focus groups, revealed a qualitative model for breakfast behaviors. This model, represented in Figure 1, consists of three domains relating to breakfast behaviors, and the internal and external factors which influence breakfast behaviors. A range of breakfast behaviors were reported including, regular and later breakfast consumption, breakfast skipping, double breakfasting, and eating breakfast on the way to school. Internal influencers of breakfast behaviors included sociocultural beliefs, views and attitudes about the breakfast meal, including the importance of breakfast, and breakfast at home vs breakfast at school. External influencers of breakfast behaviors included socioeconomic factors such as poverty, food insecurity, weekends and school holidays, work and educational commitments, and multiple or young children. It was considered that the free school breakfast scheme alleviated a number of barriers to breakfast consumption, particularly socioeconomic factors relating to poverty, food insecurity, work/educational commitments, and family structures.

The reported high levels of poverty, food insecurity, and use of food banks, reported within the community that this study was undertaken, is an area of concern, particularly in light of a recent review into household food security in the UK, which contends that accessing food aid is often a last resort when parents/carers have exhausted all other strategies (31). Furthermore, a recent report, into food poverty in the UK, shows significant increases in food bank usage between the period from 2010 to 2013, and estimates further increases in usage due to ongoing pressures on household incomes (32). According to this report, the UK’s poorest households were increasingly unable to maintain a healthy balanced diet, with accounts of individuals reducing food consumption to the point of malnutrition and children arriving to school hungry (32). Another recent large scale UK based study, on the impact of hunger in the classroom, reported an estimated 2.4 children in every class in England, and Wales were arriving to school hungry at least once a week, with concerns about the impact on learning and loss of learning time (33). Promisingly, in this study, it was considered that the council’s universal free school breakfast scheme contributed to the mitigation of food insecurity for families on low incomes, through the provision of a free breakfast for all children at school. Comparatively, a large scale US based study also found that school breakfast schemes had the potential to alleviate food-related concerns for families at-risk of food insecurity; but, however did not necessarily alleviate food insecurity once the family was experiencing high levels of deprivation (34). Further research may be therefore necessitated to identify the impacts of various school breakfast models on differing levels poverty and food insecurity.

Moreover, food insecurity, specific to weekends and school holidays, was reported in this study as a key concern. It was perceived that families were experiencing amplified food insecurity during these periods, due to the additional costs for food usually provided at school, and in the case of working parents/carers, childcare costs. Additionally, school staff reported they had organized food assistance, via local food banks, for a minority of families in the past. These findings lend support to those from a recent review into household food security and food aid in the UK (31), which reported a rise in food banks during school holidays. According to the review, an increasing number of parents/carers were experiencing difficulty in providing sufficient food during school holidays, particularly those from low income families usually in receipt of free school meals and breakfast club support (31). It has been acknowledged that the issue of food poverty is often worsened for families during the school holidays, when free school meals and in some cases free school breakfast, milk, and fruit are not available to children (35). For those who rely heavily on these food sources, the implication of the absence of this food source over the school holidays is of great concern (35). A large scale UK based study with teachers and parents reported that an estimated one in eight children may not be getting enough to eat during the school holidays, with reports from teachers of children returning to school after the school holidays having visibly lost weight and/or showing declines in readiness to learn (36).

Further socioeconomic factors in this study, which were perceived to negatively influence breakfast consumption, included: work and education educational commitments, household and family responsibilities, and multiple or very young children, each of which, it was supposed, contributed to rushed morning routines. Previous research into UK school breakfast clubs reported that rushed and chaotic mornings were cited as reasons for children not being offered breakfast in households where there is food available (37), and associations have also been suggested between rushed morning routines and breakfast skipping in children and adolescents (38–40) Encouragingly, in this study, it was perceived among participants that free school breakfast eased morning routines for families with additional responsibilities and commitments. These findings lend support to a previous qualitative pilot evaluation of the universal free school breakfast scheme, which reported perceptions that free school breakfast eased rushed morning routines and contributed to a more positive and calmer start to the school day for children (41).

Moreover, it is apparent, from the findings in this study, that certain sociocultural beliefs may act as barriers to school breakfast participation. For example, beliefs that the home environment is a superior to the school environment for consuming breakfast, parents’/carers’ evident lack of confidence in the free school breakfast scheme, and parents’/carers’ reluctance to relinquish parental control over their children’s breakfast consumption. These issues potentially necessitate a more open and transparent dialog between families, schools and other stakeholders to increase participation, and parents’/carers’ knowledge of and confidence in the free school breakfast scheme. The prevalence of the belief that breakfast was the most important meal of the day and general positive attitudes toward breakfast in general in this study, particularly in respect to children consuming breakfast, is encouraging in light of identified associations between positive attitudes toward breakfast and increased consumption of healthy foods in the morning (42). These positive attitudes toward breakfast, among participants in this study, potentially provide a positive platform in gaining increased parental support for the universal free school breakfast scheme.

Additional barriers to regular and early breakfast consumption, reported in this study, included a perceived lack of appetite in mornings, sometimes due to overeating the previous evening. These findings lend support to previous research, which has found associations between a reported lack of appetite and breakfast skipping (38). Parents/carers had concerns about the impact of their breakfast behaviors, i.e., eating breakfast later and skipping breakfast, on their children, and the potential of children modeling these perceived “negative” breakfast behaviors. These concerns may be valid in light of previous research, which has found associations between parental breakfast consumption and adolescent breakfast consumption (8, 43). Thus, perhaps, social health interventions, which are aiming to positively influence children’s dietary behaviors, should pay consideration to the significant role that parents/carers may have in influencing positive change to children’s diets, and thereby aim to foster more open dialog with parents/carers.

In this study, breakfast skipping was suspected as a method of weight control in a minority of older primary school female children. These findings provide support to previous research, which has suggested an increased prevalence of breakfast skipping among adolescent girls (44). Associations have also been found between breakfast skipping and body dissatisfaction, weight control behaviors (44), and eating disorder (45). The perceived detrimental impact of breakfast skipping on children’s learning, in this study, also lends support to previous research, which has reported associations between breakfast omission and more rapid declines in cognition across the school morning (13). Furthermore, it has been suggested that cognitive functioning is more vulnerable to the effects of short-term hunger in undernourished children (46, 47), and food insecurity may have detrimental effects on attainment (48). Therefore, by alleviating short-term hunger, universal free school breakfast may thus have the potential to improve children’s cognitive functioning, particularly in socioeconomically deprived areas with high levels of food insecurity. However, while the findings in this study lend support to the wider research findings, more research is necessitated into the impacts of school breakfast on acute cognitive functions.

On the contrasting theme of children overeating across the morning and instances of double breakfasting, a recent unpublished US based study, examining the associations between double breakfasting, i.e., consuming breakfast at home and school, with levels of physical activity and weight outcomes, reported that adolescent males were more likely to consume breakfast at home and school. The study also found a positive relationship between school breakfast participation and BMI in adolescent girls, but no relationship in adolescent males. In both sexes, school breakfast participation was not associated with the probability of being overweight^1^. Moreover, previous US based studies have found associations between school breakfast participation and reduced BMI (40, 49), and it has been suggested that school breakfast is a valuable tool in reducing obesity (50). In this study, double breakfasting was seemingly more likely to occur in environments where breakfast was served in the classroom at the start of the formal school day, and less so where breakfast was served in a canteen or dining hall before the start of the school day. Perhaps, this was due to children electing to attend school breakfast before the start of the school day, as opposed to breakfast in the classroom, where all children are typically in attendance whether they are consuming school breakfast or not. This study provides original insights into the issue of double breakfasting, specific to the provision of a universal free school breakfast. However, more research is necessitated to determine the potential effects of double breakfasting, on long-term obesity epidemics in deprived communities, particularly as breakfast provision in schools is becoming increasingly prevalent in the UK.

While the findings from the current study offer an original qualitative insight into breakfast behaviors, the barriers to breakfast consumption and school breakfast, the study is not without limitations. At the time of the study, the school breakfast scheme had been established for 1½ years, which placed limitations on the study design, meaning it was not possible to incorporate pre and post intervention measures. A further limitation with this research was the use of a sample from a small number of schools located in one area of the UK, thereby limiting the generalizability of the findings. However, the aim of this study was not to infer causality or wide ranging generalization, but instead to present an account of stakeholders’ experiences and perceptions of a universal free school breakfast scheme, within a community experiencing high levels of socioeconomic deprivation. Further research is necessitated, particularly at a national level in the UK, in order to gain a more comprehensive knowledge into the effectiveness of school breakfast, in the context of outcomes relating to the child, school, family, and wider community.

## Conflict of Interest Statement

The authors declare that the research was conducted in the absence of any commercial or financial relationships that could be construed as a potential conflict of interest.

## Footnotes

^1^Vargas AJ. Effect of School Breakfast Programs on the prevalence of breakfast skipping, double-dipping, and obesity among adolescents: a time use perspective. Paper presented at the 2015 American Social Science Association Meeting, Boston, (2015) (Unpublished).

## References

[1] An Audit of School Breakfast Club Provision in the UK [Online]. Kellogg’s (2014). Available from: http://www.kelloggs.co.uk/

[2] Dimbleby H Vincent J School Food Plan [Online]. Children’s Food Trust (2013). Available from: http://www.schoolfoodplan.com/plan/

[3] Gordon D Mack J Lansley S Main G Nandy S Patsios D The Impoverishment of the UK – PSE UK First Results: Living Standards [Online]. Poverty and Social Exclusion Team (2013). Available from: http://www.poverty.ac.uk/pse-research/pse-uk-reports

[4] Rampersaud GC Benefits of breakfast for children and adolescents: update and recommendations for practitioners. Am J Life Med (2009) 3(2):86–103. 10.1177/1559827608327219

[5] Potamites E Gordon A Children’s Food Security and Intakes from School Meals [Online]. Department of Agriculture, Economic Research Service (2010). Available from: http://naldc.nal.usda.gov/download/42320/PDF

[6] Currie C Zanotti C Morgan A Currie D de Looze M Roberts C Social Determinants of Health and Wellbeing Among Young People: Health Behaviour in School Aged Children (HBSC) Study [Online]. World Health Organisation (2010). Available from: http://www.euro.who.int/__data/assets/pdf_file/0003/163857/Social-determinants-of-health-and-well-being-among-young-people.pdf

[7] Vereecken C Dupuy M Rasmussen M Kelly C Nansel TR Sabbah HA Breakfast consumption and its socio-demographic and lifestyle correlates in schoolchildren in 41 countries participating in the HSBC study. Int J Pub Health (2009) 54(2):180–90. 10.1007/s00038-009-5409-5 19639257 PMC3408388

[8] Keski-Rahkonen A Kaprio J Rissanen A Virkkunen M Rose RJ. Breakfast skipping and health-compromising behaviors in adolescents and adults. Eur J Clin Nutr (2003) 57:842–53. 10.1038/sj.ejcn.1601618 12821884

[9] Huang CJ Hu HT Fan YC Liao YM Tsai PS. Associations of breakfast skipping with obesity and health-related quality of life: evidence from a national survey in Taiwan. Int J Obes (2010) 34:720–5. 10.1038/ijo.2009.285 20065977

[10] Elgar FJ Roberts C Moore L Tudor-Smith C. Sedentary behaviour, physical activity and weight problems in adolescents in Wales. Pub Health (2005) 119:518–24. 10.1016/j.puhe.2004.10.011 15826893

[11] Revicki D Sobal J DeForge B. Smoking status and the practice of other unhealthy behaviours. Fam Med J (1991) 23:361–4. 1884931

[12] Utter J Scragg R Mhurchi C Schaaf D. At-home breakfast consumption among New Zealand children: associations with body mass index and related nutrition behaviors. J Am Diet Ass (2007) 107:570–6. 10.1016/j.jada.2007.01.010 17383261

[13] Wesnes KA Pincock C Scholey A. Breakfast is associated with enhanced cognitive function in schoolchildren. An internet based study. Appetite (2012) 59(3):646–9. 10.1016/j.appet.2012.08.008 22902600

[14] Defeyter MA Russo R. The effect of breakfast consumption on adolescents academic performance and mood. Front Hum Neuro (2013) 7:789. 10.3389/fnhum.2013.00789 24312043 PMC3834293

[15] Wesnes KA Pincock C Richardson D Helm D Hails S. Breakfast reduces declines in attention and memory over the morning in schoolchildren. Appetite (2013) 41:329–31. 10.1016/j.appet.2003.08.009 14637332

[16] Widenhorn-Müller K Hille K Klenk J Weiland U. Influence of having breakfast on cognitive performance and mood in 13- to 20-year-old high school students: results of a crossover trial. Pediatrics (2008) 122(2):279–84. 10.1542/peds.2007-0944 18676544

[17] Kleinman RE Hall S Green H Korzec-Ramirez D Patton K Paganoe ME Diet, breakfast, and academic performance in children. Ann Nutr Metab (2002) 46(1):24–30. 10.1159/000066399 12428078 PMC3275817

[18] Simpson D The impact of breakfast clubs on pupil attendance and punctuality. Res Ed (2001) 66:76–83. 10.7227/RIE.66.7

[19] Richter LM Rose C Griesel RD Cognitive and behavioural effects of a school breakfast. S Afr Med J (1996) 87(1):93–100. 9180808

[20] Bro RT Shank LL McLaughlin TF Williams RL Effects of a breakfast program on on-task behaviours of vocational high school students. J Ed Res (1996) 16(3):1–8. 10.1300/J019v16n03_01

[21] Benton D Maconie A Williams C. The influence of the glycaemic load of breakfast on the behaviour of children in school. Physio Behav (2007) 92(4):717–24. 10.1016/j.physbeh.2007.05.065 17617427

[22] Mhurch CN Gorton D Turley M Jiang Y Michie J Maddison R Effects of a free school breakfast programme on children’s attendance, academic achievement and short term hunger: results from a stepped wedge, cluster randomised control trial. Epid Com Health (2012) 3:257–64. 10.1136/jech-2012-201540 23043203 PMC3582067

[23] Acham H Kikafunda J Malde M Oldewage-Theron W Egal A. Breakfast, midday meals and academic achievement in rural primary schools in Uganda: implications for education and school health policy. Food Nutr Res (2012) 56. 10.3402/fnr.v56i0.11217 22347147 PMC3280795

[24] Shemilt I Harvey I Shepstone L Swift L Reading R Mugford M A national evaluation of school breakfast clubs: evidence from a cluster randomized controlled trial and an observation analysis. Ch Care Health Dev (2004) 30(5):413–27. 10.1111/j.1365-2214.2004.00453.x 15320919

[25] Bernstein LS McLaughlin JE Crepinsek MJ Evaluation of the School Breakfast Pilot Programme: Summary of Findings From the First Year of Implementation [Online]. Department of Agriculture, Food and Nutrition (2002). Available from: http://www.fns.usda.gov/evaluation-school-breakfast-program-pilot-project-findings-first-year-implementation

[26] Chang SM Walker SP Himes J Grantham-McGregor SM Effects of breakfast on classroom behaviour in rural Jamaican schoolchildren. Food Nutr Bull (1996) 17:248–57.

[27] Indices of Deprivation [Online]. Office for National Statistics, Department for Communities and Local Government, Homes and Communities Agency (2013). Available from: http://neighbourhood.statistics.gov.uk/dissemination/.

[28] Ajzen I The theory of planned behavior. Org Behav Hum Dec Proc (1991) 50:179–211. 10.1016/0749-5978(91)90020-T

[29] Strauss A Corbin J Basics of Qualitative Research: Grounded Theory Procedures and Techniques. Newbury Park, CA: Sage (1990). 270 p.

[30] Guyatt G Rennie D Meade MO Cook DJ Users’ Guides to the Medical Literature: A Manual for Evidence Based Clinical Practice. 3rd rev ed Chicago, IL: McGraw-Hill (2015).

[31] Lambie-Mumford H Crossley D Jensen E Verbeke M Dowler E Household Food Security in the UK: A Review of Food Aid Final Report [Online]. Department for Environment, Food & Rural Affairs (2014). Available from: https://www.gov.uk/government/uploads/system/uploads/attachment_data/file/283071/household-food-security-uk-140219.pdf

[32] Gordon D Mack J Lansley S Main G Nandy S Patsios D The Impoverishment of the UK PSE UK First Results: Living Standards [Online]. Economic and Social Research Council (2013). Available from: http://www.poverty.ac.uk/pse-research/pse-uk-reports

[33] Kellogg’s. A Lost Education: The Reality of Hunger in the Classroom [Online]. You Gov Plc (2013). Available from: http://pressoffice.kelloggs.co.uk/index.php?s=20295&item=122412

[34] Bartfeld JS Ahn H. The School Breakfast Program strengthens household food security among low-income households with elementary school. J Nutr (2011) 141(3):470–5. 10.3945/jn.110.130823 21228262

[35] Rai S Food Poverty: School Holidays and the Wider Impact [Online]. Northern Housing Consortium (2015). Available from: http://www.northern-consortium.org.uk/assets/Policy%20Documents/childrenandyoungpeople/food-poverty-briefing-w-kelloggs.pdf

[36] School Holidays Leave Kids Hungry for Three Meals A Day. [Online]. Kellogs & Trussell Trust (2014). Available from: http://www.trusselltrust.org/holiday-hunger

[37] Harrop A Palmer G Improving Breakfast Clubs: Lessons From The Best [Online]. New Policy Institute (2002). Available from: http://npi.org.uk/publications/children-and-young-adults/improving-breakfast-clubs-lessons-best/

[38] Reddan J Wahlstrom K Reicks M. Children’s perceived benefits and barriers in relation to eating breakfast in schools with or without universal school breakfast. Nutr Educ Behav (2002) 34(1):47–52. 10.1016/S1499-4046(06)60226-1 11917671

[39] Bruening M Larson N Story M Neumark-Sztainer D Hannan P. Predictors of adolescent breakfast consumption: longitudinal findings from project EAT. J Nutr Educ Behav (2011) 43(5):390–5. 10.1016/j.jneb.2011.02.016 21906551

[40] Wahba SA Mekawy AA Ahmed RT Mohsen WA Breakfast skipping and dietary adequacy of primary school children in Cairo. J App Sci Res (2006) 2:51–7.

[41] Graham P Russo R Blackledge J Defeyter MA Breakfast and beyond: the dietary, social and practical impacts of a universal free school breakfast scheme in the North West of England, UK. Int J Soc Agr Food (2014) 3:261–74.

[42] Tapper K Murphy S Moore L Clark R. Development of a scale to measure 9-11-year olds’ attitudes towards breakfast. Eur J Clin Nut (2008) 62(4):511–8. 10.1038/sj.ejcn.1602735 17375113

[43] Pearson S Biddle JH Gorely T. Family correlates of breakfast consumption among children and adolescents. A systematic review. Appetite (1998) 52(1):1–7. 10.1016/j.appet.2008.08.006 18789364

[44] Shaw ME. Adolescent breakfast skipping: an Australian study. Adolescence (1998) 33(132):851–61. 9886013

[45] Fernández-Arandaa F Kruga I Graneroc R Ramóna JM Badiaa A Giméneza L Individual and family eating patterns during childhood and early adolescence: an analysis of associated eating disorder factors. Appetite (2007) 49(2):476–85. 10.1016/j.appet.2007.03.004 17467116

[46] Powell CA Walker SP Chang SM Grantham-McGregor SM. Nutrition and education: a randomized trial of the effects of breakfast in rural primary school children. Am J Clin Nut (1998) 68(4):873–9. 9771865 10.1093/ajcn/68.4.873

[47] Grantham-McGregor SM Chang S Walker SP. Evaluation of school feeding programs: some Jamaican examples. Am J Clin Nutr (1998) 67(4):785–9. 9537629 10.1093/ajcn/67.4.785S

[48] Winicki J Jemison K Food insecurity and hunger in the kindergarten classroom: its effect on learning and growth. Cont Econ Policy (2003) 21:145–57. 10.1093/cep/byg001

[49] Gleason PM Dodd AH. School breakfast program but not school lunch program participation is associated with lower body mass index. J Am Diet Assoc (2009) 109(2):118–28. 10.1016/j.jada.2008.10.058 19166666

[50] Millimet DL Tchernis R Husain M School Nutrition Programs and the incidence of childhood obesity. J Hum Res (2009) 45:3 10.3386/w14297

